# Author Correction: Cytoskeleton remodeling induced by SMYD2 methyltransferase drives breast cancer metastasis

**DOI:** 10.1038/s41421-024-00661-4

**Published:** 2024-03-26

**Authors:** Alexandre G. Casanova, Gael S. Roth, Simone Hausmann, Xiaoyin Lu, Ludivine J. M. Bischoff, Emilie M. Froeliger, Lucid Belmudes, Ekaterina Bourova-Flin, Natasha M. Flores, Ana Morales Benitez, Tourkian Chasan, Marcello Caporicci, Jessica Vayr, Sandrine Blanchet, Francesco Ielasi, Sophie Rousseaux, Pierre Hainaut, Or Gozani, Muriel Le Romancer, Yohann Couté, Andres Palencia, Pawel K. Mazur, Nicolas Reynoird

**Affiliations:** 1 Grenoble Alpes University, CNRS UMR 5309, INSERM U 1209, Institute for Advanced Biosciences, Grenoble, France; 2 Clinique Universitaire d’Hépato-gastroentérologie et Oncologie digestive, CHU Grenoble Alpes, Grenoble, France; 3 Department of Experimental Radiation Oncology, The University of Texas MD Anderson Cancer Center, Houston, TX USA; 4 Grenoble Alpes University, CEA, INSERM, UA13 BGE, CNRS CEA, FR2048 Grenoble, France; 5 Department of Biology, Stanford University, Stanford, CA USA; 6 Université de Lyon, Centre de Recherche en Cancérologie de Lyon, Inserm U1052, CNRS UMR5286 Lyon, France

**Keywords:** Breast cancer, Metastasis, Methylation, Lamellipodia, Proteomic analysis

Correction to: *Cell Discovery* (2024) 10:12

10.1038/s41421-023-00644-x published online 31 January 2024

In the initial publication of this article, we unintentionally misplaced an inaccurate image in Fig. 7a for the representative mice’s lungs H&E staining at day 35 post-injection of engineered MDA-MB-231 cells with doxycyclin treatment rescued with K334A BCAR3. The correct Fig. 7 is displayed as below. In addition, we stated in the methods section ‘Identification of methyl-sensitive binders’ that HeLa cells were used while the related experiments were performed with MDA-MB-231 cells.

**Fig. 7:**
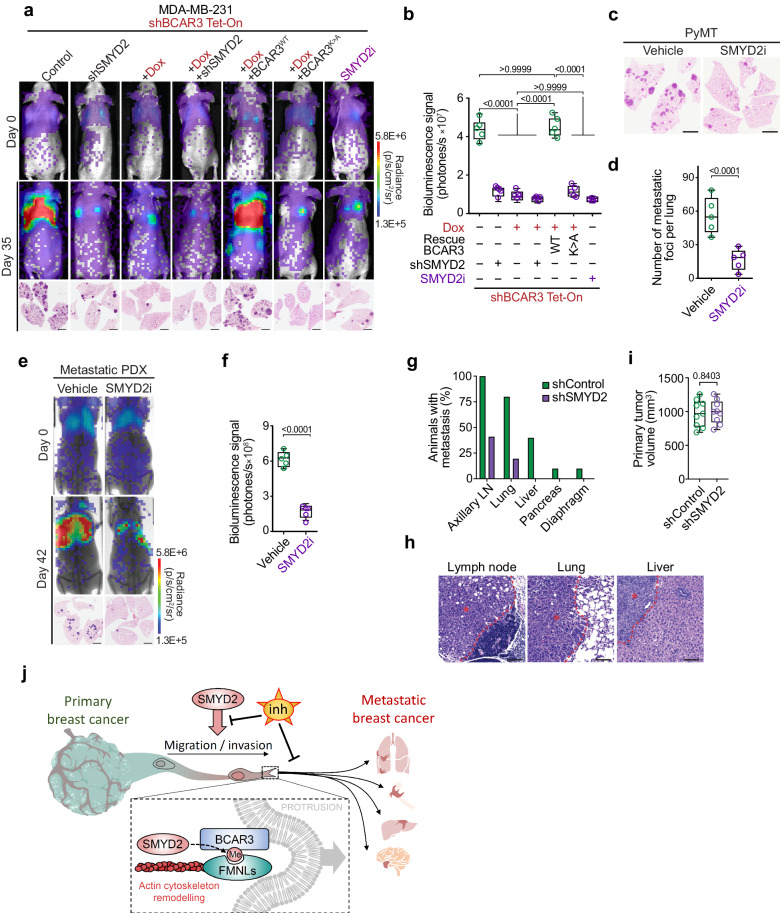
The SMYD2-BCAR3-FMNLs axis drive breast cancer metastasis. **a** Representative bioluminescence imaging of animals at the time of intravenous transplantation (day 0) and 35 days post-injection of MDA-MB-231 cells with indicated engineering. Lower panel, representative HE staining of the lungs at day 35. Representative of *n* = 5 mice for each experimental group. Scale bars, 3 mm. **b** Quantification of bioluminescence signal corresponding to metastatic cancer growth in animals as in (**a**) at day 35. Representative of *n* = 5 mice for each experimental group. *P*-values were calculated by ANOVA with Tukey’s testing for multiple comparisons. **c, d** Representative HE staining (**c**) and quantification (**d**) of metastatic foci in the lungs of *PyMT* mice treated with SMYD2i inhibitor or vehicle (control) at 12 weeks of age. Representative of *n* = 5 mice for each experimental group. *P*-value was calculated by two-tailed unpaired *t*-test. **e, f** Representative bioluminescence visualization (**e**) and signal quantification (**f**) of breast cancer cells obtained from patient-derived xenografts and intravenously transplanted into recipient NSG mice and treated with SMYD2i inhibitor or vehicle (control). Lower panel representative HE staining of metastatic foci in the lungs at day 42. Representative of *n* = 5 mice for each experimental group. Scale bars, 3 mm. *P*-value was calculated by two-tailed unpaired *t*-test. **g** Quantification of the percentage of animals bearing macro-metastasis in each organ observed at the time of necropsy (7 weeks post injection) following orthotopic implantation of control or SMYD2-depleted MDA-MB-231 into mammary fat pad (*n* = 10 mice for each experimental group). **h** HE staining of the representative metastases observed in indicated tissues of the control MDA-MB-231 cells injected animals (as in **g**); asterisk indicates tumor, dashed line marks tumor margins. **i** Quantification of the primary tumor volume of orthotopically implanted MDA-MB-231 cells at the time of necropsy (7 weeks post injection). *P*-value was calculated by two-tailed unpaired *t*-test. **j** SMYD2 regulates breast cancer cells motility and metastasis spreading through methylation of BCAR3 and recruitment of FMNLs to protrusive membrane structures such as lamellipodia. FMNLs localization at nascent lamellipodia enables nucleation and elongation of actin filaments and generates the force required for cell migration. Hence, upregulation of the SMYD2-BCAR3-FMNLs pathway, commonly observed in the malignant breast cancer cell, promotes metastatic spread. Pharmacological inhibition of SMYD2 enzymatic activity in pre-clinical in vivo animal models can efficiently prevents breast cancer’s ability to metastasize. In all box plots, the center line indicates the median, the box marks the 75th and 25th percentiles and whiskers: min. to max. values.

These corrections neither affect the results nor the conclusion of this work. We apologize for any inconvenience these inadvertent errors might have caused.

